# Effects of tracking linkage self-management mode on the compliance of prenatal examinations and delivery modes in primiparas

**DOI:** 10.1097/MD.0000000000038494

**Published:** 2024-06-28

**Authors:** Yufan Yuan, Xiaojing Zhao, Zhengli Kang, Xiufang He, Xianfang Song

**Affiliations:** a Department of Nursing, Shijiazhuang Obstetrics and Gynecology Hospital Shijiazhuang, Hebei, China; b Department of Maternity, Shijiazhuang Obstetrics and Gynecology Hospital Shijiazhuang, Hebei, China; c Department of International, Shijiazhuang Obstetrics and Gynecology Hospital Shijiazhuang, Hebei, China.

**Keywords:** compliance, delivery mode, prenatal examination, primipara, tracking linkage self-management mode

## Abstract

To explore the effects of tracking linkage self-management mode on the compliance of prenatal examinations and delivery modes in primiparas. A total of 270 primiparas undergoing prenatal examinations in Shijiazhuang Obstetrics and Gynecology Hospital were enrolled for prospective study between January 2021 and January 2022. They were divided into control group and observation group, 135 cases in each group. The control group was given routine management mode, while observation group was given tracking linkage self-management mode. All were intervened till discharge. The compliance (time and frequency of prenatal examinations), cognition of prenatal examinations, score of exercise of self-care agency scale, self-rating anxiety scale and self-rating depression scale, delivery modes and the occurrence of neonatal adverse outcomes were compared between the 2 groups. After intervention, total compliance rate of prenatal examinations in observation group was higher than that in control group (84.44% vs 72.59%) (*P* < .05). The scores of pregnancy care, genetic diseases counseling, prevention of birth defects and reasonable nutrition during pregnancy in observation group were higher than those in control group (*P* < .05), scores of health cognition, self-care skills, self-care responsibility and self-concept were higher than those in control group (*P* < .05), scores of self-rating anxiety scale and self-rating depression scale were lower than those in control group (*P* < .05), natural delivery rate was higher than that in control group (85.93% vs 74.81%) (*P* < .05), and incidence of neonatal adverse outcomes was lower than that in control group (0.74% vs 5.93%) (_Fisher exact probability_ = 0.036). The application of tracking linkage self-management mode can significantly improve cognition to prenatal examinations, improve compliance of prenatal examinations and self-care ability, relieve anxiety and depression, increase natural delivery rate and reduce the incidence of neonatal adverse outcomes in primiparas.

## 1. Introduction

With the continuous improvement of people’s health awareness, many women will conduct prenatal examinations before pregnancy to assess their own health status, so as to prepare for the birth of a healthy fetus. The purpose of prenatal examination is to clarify the health status of pregnant women and fetuses, to detect and treat pregnancy complications and complications as soon as possible, to correct abnormal fetal position or to detect congenital diseases of the fetus in a timely manner, and to provide a strong basis for subsequent treatment.^[1]^ At present, most of the implementations in my country are still the traditional prenatal examination mode, that is, 12 to 14 regular prenatal examinations. There are great differences in the protocols of prenatal examinations in different regions and hospitals in my country, and the compliance of different groups of people with prenatal examinations is unbalanced.^[2]^ In recent years, follow-up self-management education, as a continuous service mode for patients who need a longer nursing cycle, can improve and maintain physical health through their own behaviors.^[3]^ Previous studies^[4]^ reported that the impact of follow-up self-management education on postpartum women after discharge, but rarely reported the impact of this management model on maternal prenatal examination. The impact on obstetric inspection compliance and delivery mode provides a basis for better perfecting the prenatal inspection system suitable for my country’s national conditions.

## 2. Materials and methods

### 2.1. General information

The study was approved by the Ethics Committee of the Shijiazhuang Obstetrics and Gynecology Hospital(2023 No.054). A prospective study of 270 primiparas who underwent obstetric examination in Shijiazhuang Obstetrics and Gynecology Hospital from January 2021 to January 2022 was carried out. They were divided into control group and observation group according to different nursing methods, with 135 cases in each group. Inclusion criteria: all were singleton pregnancies, and the first obstetric examination was 12 weeks pregnant; no abnormal pregnancy such as gestational diabetes, gestational hypertension, abnormal fetal position; inform pregnant women and their families about the content of this study, all willing to cooperate, and in Informed consent was signed and confirmed. Exclusion criteria: those with mental disorders or unable to communicate easily and effectively; all have undergone obstetric examinations in our hospital, and the data of each obstetric examination are complete and can be studied; have the conditions for childbirth. Control group (n = 135): average age (27.41 ± 3.01) years old (range 20–36 years old); education level: 21 cases below high school, 66 cases with high school and college education, and 48 cases with bachelor degree or above; monthly household income: 20 cases below 8000 yuan, 70 cases between 8000 and 12,000 yuan, and 45 cases above 12,000 yuan. Observation group (n = 135): average age (27.46 ± 2.88) years old (range 20–34 years old); education level: 19 cases below high school, 67 cases with high school and college education, 49 cases with bachelor degree or above; monthly household income: 19 cases below 8000 yuan, 73 cases between 8000 and 12,000 yuan, and 43 cases above 12,000 yuan. There was no significant difference in the basic data between the 2 groups (*P* > .05), which can be compared and analyzed. This study complies with the relevant ethical guidelines of the Declaration of Helsinki.

### 2.2. Methods

Pregnant women in both groups followed a routine obstetric checkup cycle at 12, 16, 20, 24, 28, 30, 32, 34, 36, 37, 38, 39, and 40 weeks of pregnancy Take a pregnancy test. The control group adopted the routine management mode to popularize the concept and importance of prenatal examinations to the mothers and their families, to inform them about the knowledge of physiological hygiene, life and nutrition during pregnancy, and to inform them of the problems that should be paid attention to before and after childbirth and the common sense of normal childbirth. The observation group adopted the tracking and linkage self-management mode, and the details are as follows:establish a tracking and linkage self-management education group, whose members include 1 physician, 1 nurse with >5 years of experience and 2 midwifery nurses;make a maternal self-management notice. All members of the tracking and linkage self-management education team participated in the discussion and production, and the contents of the notice included the relevant knowledge that should be paid attention to during pregnancy, such as rational drug use during pregnancy, nutrition during pregnancy, health care during pregnancy, genetic diseases and birth defects of newborns. During the first checkup of the puerpera, a maternal self-management notice will be issued to her and her family members, and the doctor will explain the content and answer their doubts word by word, and instruct the family members to have someone to accompany the pregnant woman for at least 3 hours during the day. Live broadcast of prenatal education. A live prenatal education event will be organized once a month, and the physician will decompose the matters that pregnant women need to pay attention to at each stage, including the advantages and disadvantages of natural childbirth and cesarean section, and instruct pregnant women to take appropriate outdoor and social activities through live broadcast. Be active and stay in the mood. Establish real-time WeChat contacts. Group members will establish real-time WeChat communication with pregnant women or their family members, and timely answer relevant questions in the daily life of pregnant women and their family members; Voice communication with pregnant women and their families every weekend to understand the physical and psychological state of pregnant women, correct unscientific and unreasonable living habits of mothers and their families; and supervise pregnant women with the assistance of their families so that they can consciously abide by them Precautions for pregnant women to form a self-management model; Pregnant women should be notified in advance to go to the hospital for antenatal checkups as scheduled within 1 week of the time of the maternity checkup.

### 2.3. Observation indicators

Record the obstetric examination time and the number of obstetric examinations of all included pregnant women, and compare the obstetric examination compliance. All included pregnant women underwent prenatal examination cognitive assessment after 4 weeks of intervention in different management modes. The hospital-made scale was used for scoring. The content of the scale includes 4 dimensions: genetic disease consultation, health care during pregnancy, reasonable nutrition during pregnancy, and prevention of birth defects. Each dimension is scored from 0 to 100 points. The higher the score means the better the cognition of obstetric examination. The scale was evaluated by pre-experiment, and the consistency reliability Cronbach alpha was 0.84. All pregnant women were evaluated for self-care ability before intervention and after 4 weeks of intervention in different management modes. The exercise of self-care agency scale (ESCA)^[5]^ is used to score, and the scale content includes health cognition (0–68 points), self-care skills (0–48 points), and self-care responsibility (0–24 points), self-concept (0–32 points) 4 dimensions, the higher the score, the stronger the self-protection ability, the Cronbach *α* of the scale’s consistency reliability is 0.86. All included pregnant women were evaluated for prenatal anxiety and depression before the intervention and at 36 weeks of pregnancy with different management mode intervention values. The self-rating anxiety scale (SAS) and the self-rating depression scale (SDS) were used for evaluation. The total score of the 2 scales was 0 to 80. The higher the score indicates the more severe the anxiety and depression.^[6]^ The scale Cronbach alpha was 0.93, 0.95. Record the final delivery mode of all included pregnant women, and compare the vaginal delivery rate between the observation group and the control group. To record the comparison of adverse macrosomia outcomes between the 2 groups, including low birth weight infants, macrosomia asphyxia, and jaundice.

### 2.4. Statistical methods

SPSS 22.0 statistical software was used for data analysis, and (x ®±s) was used to represent the measurement data that met the normal distribution and the variance was equal, and the *t* test was performed; The enumeration data were expressed by the number of cases or rates, and *χ*^2^ or Fisher exact test was performed; *P* < .05 indicated a statistically significant difference.

## 3. Results

### 3.1. Comparison of obstetric examination time and number of obstetric examinations between the 2 groups of pregnant women

**Figure 1.**
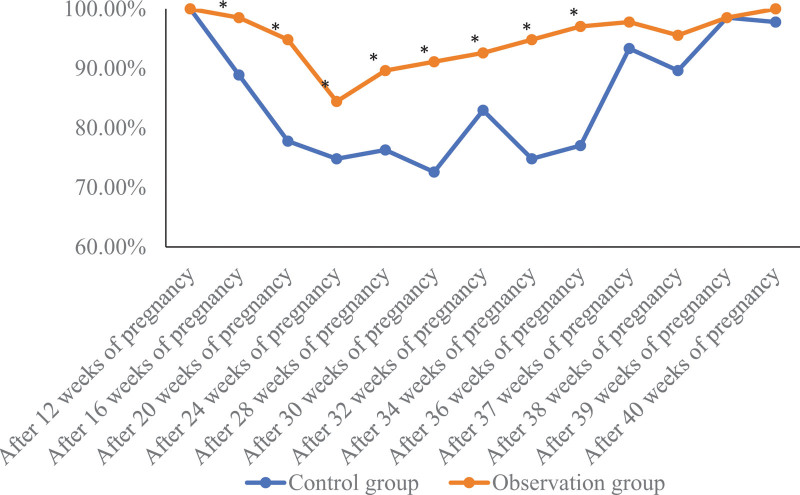
Comparison of obstetric examination time and number of obstetric examinations between 2 groups of pregnant women. Compared with the control group, * means *P* < .05.

The obstetric inspection compliance rate of pregnant women in the 2 groups in the early and later stages of pregnancy was high. In the second trimester of pregnancy, the rate of scheduled obstetric inspection of pregnant women in the observation group was higher than that in the control group. The complete obstetric inspection compliance in the observation group was 84.44% (114/135), which was higher than that in the control group, 72.59% (98%)/135), the difference was statistically significant (*χ*^2^ = 5.621, *P* = .018). As shown in Figure 1.

### 3.2. Comparison of the cognition of prenatal examination between the 2 groups of pregnant women

**Figure 2.**
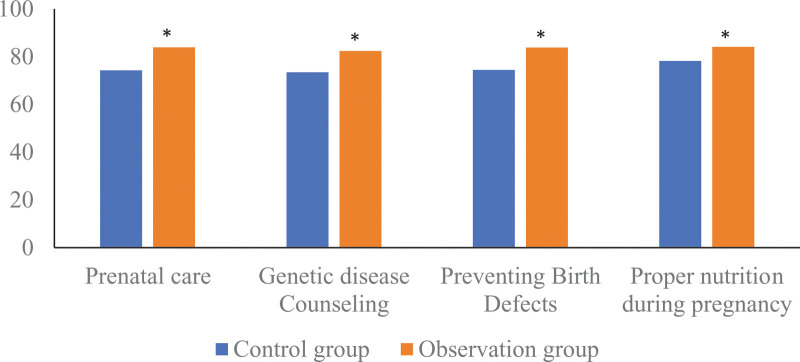
Comparison of the cognition of prenatal examination among 2 groups of pregnant women. Compared with the control group, * means *P* < .05.

After intervention, pregnant women in the observation group had significantly higher cognitive scores on prenatal examination than those in the control group. The scores of the observation group on health care during pregnancy, hereditary disease counseling, prevention of birth defects, and reasonable nutrition during pregnancy were (83.90 ± 7.79) and (82.35 ± 7.70), respectively. points, (83.76 ± 7.56) points, (84.13 ± 7.98) points were higher than those of the control group (74.28 ± 9.04) points, (73.41 ± 9.25) points, (74.44 ± 9.28) points, (78.19 ± 10.74) points, the differences were statistical significance (*t* = 9.370, 8.623, 9.039, 5.165, *P* < .05). as shown in Figure 2.

### 3.3. Comparison of self-care ability of pregnant women in the 2 groups

**Figure 3.**
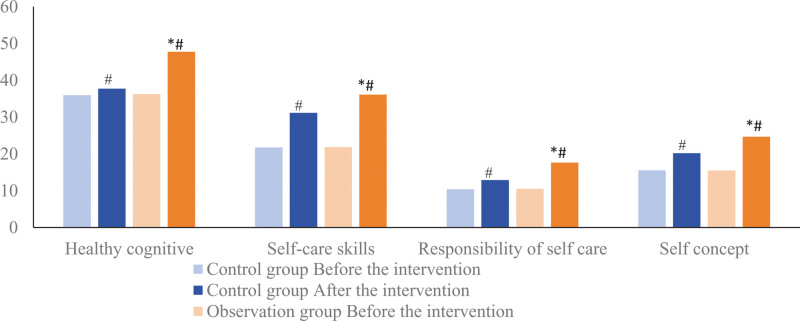
Comparison of self-care ability of pregnant women in 2 groups. Compared with the control group, * means *P* < .05; compared with the same group before intervention, # means *P* < .05.

The ESCA scores of pregnant women in the 2 groups were significantly increased after nursing intervention, and the scores of health cognition, self-care skills, self-care responsibility and self-concept of the observation group were (47.72 ± 6.21), (36.07 ± 4.09), (17.62 ± 3.24)) points and (24.67 ± 2.97) points were higher than those of the control group (37.70 ± 4.92) points, (31.12 ± 3.77) points, (12.87 ± 2.78) points and (20.18 ± 3.53) points, and the differences were statistically significant (*t* = 14.696, 10.345, 12.922, 11.314, *P* < .05). As shown in Figure 3.

### 3.4. Comparison of prenatal anxiety and depression in 2 groups of pregnant women

**Figure 4.**
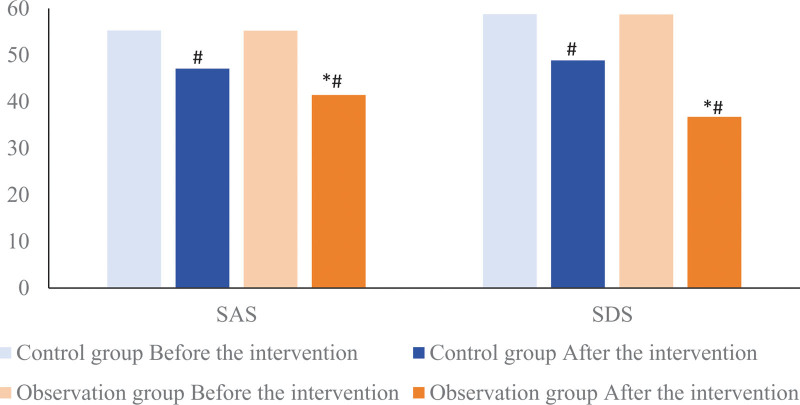
Comparison of prenatal anxiety and depression in 2 groups of pregnant women. Compared with the control group, * means *P* < .05; compared with the same group before intervention, # means *P* < .05.

The SAS and SDS scores of the 2 groups of pregnant women were significantly reduced after nursing intervention, and the SAS and SDS scores of the observation group were (41.44 ± 5.28) and (36.70 ± 4.58) points lower than those of the control group (47.10 ± 5.17) and (48.81 ± 5.28) 6.25) points, the difference was statistically significant (*t* = 8.912, 18.177, *P* < .05). As shown in Figure 4.

### 3.5. Comparison of delivery methods between the 2 groups of pregnant women

**Figure 5.**
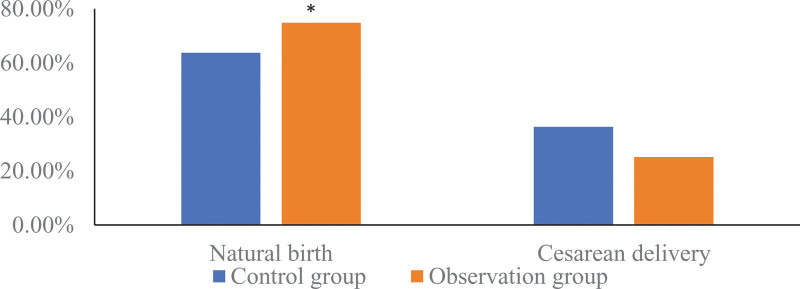
Comparison of delivery methods of 2 groups of pregnant women. Compared with the control group, * means *P* < .05.

There were 116 cases of vaginal delivery in the observation group, with a vaginal delivery rate of 85.93% (116/135); 101 cases in the control group, with a vaginal delivery rate of 74.81% (101/135); the vaginal delivery rate in the observation group was higher than that in the control group, and the difference was statistically significant (*χ*^2^ = 5.282, *P* = .022). As shown in Figure 5.

### 3.6. Comparison of adverse neonatal outcomes between the 2 groups

**Figure 6.**
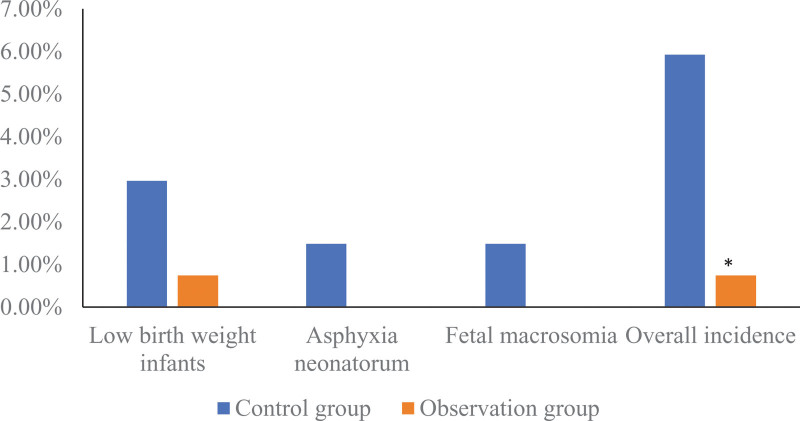
Comparison of adverse neonatal outcomes between the 2 groups. Compared with the control group, * means *P* < .05.

There was 1 low birth weight infant in the observation group, and the incidence of adverse neonatal outcome was 0.74% (1/135). The incidence rate was 5.93% (8/135); Compared with the control group, the incidence of adverse neonatal outcomes in the observation group was lower (Fisher exact probability = 0.036). As shown in Figure 6.

## 4. Discussions

With the liberalization of the 2-child policy, more and more elderly women have joined the “birth army,” so the number of birth defects is also increasing.^[7]^ Defective children often have different degrees of disability, which not only brings huge economic burden and mental pressure to the family,^[8]^ but also affects the improvement of the overall quality of our nationals. However, it is gratifying that in recent years, my country’s prenatal diagnosis technology has been developed by leaps and bounds. There are various prenatal diagnosis methods. In addition to routine chromosome analysis, gene chips and high-throughput gene sequencing technologies have been widely used. Clinically, the rate of prenatal diagnosis of fetuses with birth defects is increasing.^[9,10]^ However, due to the lack of awareness of the importance of prenatal examinations for some mothers, they are unwilling to go to the hospital for regular obstetric examinations, resulting in the birth of newborns with defects. The conventional prenatal education model can only provide oral education when pregnant women arrive at the hospital, which may not enable pregnant women to achieve a high level of self-management, and often have low obstetric compliance. Relevant research^[11]^ reported that self-management mode can effectively improve patients’ self-management ability and improve their quality of life. Based on this, this study used the tracking and linkage self-management model for the prenatal management of primipara in our hospital, and found that the effect was significant.

In this study, the complete obstetric examination compliance of pregnant women in the observation group applying the tracking and linkage self-management mode was 84.44%, which was significantly higher than that in the control group (72.59%) under the conventional management mode. The cognitive status of prenatal examinations such as reasonable nutrition during pregnancy was better than those of the control group. Previous studies^[12]^ have shown that flexible forms of pregnancy health education can effectively improve the compliance of pregnant women in Tibet. Another study^[13]^ reported that real-time health management of pregnant women can effectively improve the compliance rate of pregnant women’s obstetric examination and the rate of natural childbirth. Combined with the above research reports, this study suggests that the tracking and linkage self-management model for antenatal health management of primiparas can effectively enhance pregnant women’s awareness of the importance of antenatal examinations and improve obstetric examination compliance. The reason may be that the tracking and linkage self-management mode not only prepares the maternal self-management notice for easy viewing by pregnant women and their families at any time, but also conducts regular prenatal education, so that pregnant women can more easily recognize the importance of obstetric examinations and are more willing to accept obstetric examinations., thereby improving obstetric compliance.

Relevant studies^[14]^ show that mental health problems such as prenatal anxiety and depression are closely related to the emotional development of offspring; Another study^[15]^ showed that prenatal mental health may increase the risk of childhood asthma. Therefore, it is of great significance to implement standardized and systematic health knowledge education for primiparas to alleviate negative emotions such as anxiety and depression of pregnant women. In this study, the ESCA scores (including health cognition, self-care skills, self-care responsibility, and self-concept) of pregnant women in the observation group using the tracking and linkage self-management model were higher than those in the control group under the conventional management model; At the same time, the reduction degree of SAS and SDS scores of pregnant women in the observation group was better than that in the control group. Relevant studies^[16]^ reported that improving the lifestyle of pregnant women can effectively alleviate their negative psychological emotions during pregnancy. Combined with the above reports, this study suggests that the tracking and linkage self-management model can be used in the prenatal health management of primipara, which can improve the self-care ability of pregnant women and relieve their anxiety and depression. The reason may be that, under the tracking and linkage self-management mode, real-time attention is paid to the physical and mental health of pregnant women, and the self-management of pregnant women is supervised to form a “self-centered” management mode, so as to improve the self-care ability of pregnant women, and not have childbirth. pre-fear.

This study found that the natural delivery rate of the observation group in the observation group using the tracking and linkage self-management mode was 85.93%, which was significantly higher than that in the control group (74.81%) under the conventional management mode, and the incidence of adverse neonatal outcomes in the observation group was significantly lower than that in the control group. It is suggested that the tracking and linkage self-management mode is used for the prenatal health management of primipara, which can improve the natural birth rate of primipara and reduce the incidence of adverse neonatal outcomes. The reason may be that, under the tracking and linkage self-management mode, doctors provide professional prenatal education support, so that pregnant women realize the importance of natural childbirth, and consciously pay attention to reasonable nutrition and health care during pregnancy, so as to reduce the number of anatomical diseases caused by medical indications. uterine delivery,^[17]^ and increase the rate of vaginal delivery of mothers. The tracking and linkage self-management mode has a standardized propaganda and education process. Professional physicians regularly conduct prenatal education, and pay attention to the psychological and physiological state of the mother in real time, so that the mother can ensure the best physical quality and can carry out natural childbirth, thereby effectively reducing the cesarean section rate.. In addition, with the continuous development of medical technology, the update and iteration of technologies and equipment such as electrocardiography, ultrasonography, fetal heart monitoring, and fetal maturity examination, the efficiency of prenatal diagnosis has been significantly improved.^[18,19]^ The tracking and linkage self-management model can not only improve the obstetric inspection compliance of pregnant women, but also give pregnant women and their families correct prenatal guidance in a timely manner, so that pregnant women can avoid risk factors that lead to adverse pregnancy outcomes as much as possible, and significantly reduce the incidence of adverse neonatal outcomes.^[20]^

This study still has some limitations: the sample size of this study is too small; since we did not observe the effect of this nursing mode on female patients after delivery in this study, we will continue to extend the follow-up time to observe the effect on postpartum. We plan to conduct a multicenter study with extended follow-up to observe long-term outcomes in women.

In conclusion, the application of the tracking and linkage self-management model to primiparas can significantly improve maternal cognition of obstetric examination, improve obstetric examination compliance, improve maternal self-care ability, and relieve anxiety and depression. Increase the rate of vaginal delivery and reduce the incidence of adverse neonatal outcomes. However, this study may have certain data bias due to the single-center study, and most of them are women of childbearing age. The impact on high-risk mothers is not yet clear. In the later stage, further multicenter and large-sample trials are needed to evaluate high-risk mothers with advanced age or pregnancy diseases. A study was conducted to comprehensively explore the impact of the tracking linkage self-management model on maternal compliance with obstetric examination and pregnancy outcomes.

## Author contributions

**Conceptualization:** Yufan Yuan, Xiaojing Zhao, Zhengli Kang, Xianfang Song.

**Data curation:** Yufan Yuan, Xiaojing Zhao, Xiufang He, Xianfang Song.

**Formal analysis:** Yufan Yuan.

**Investigation:** Xiaojing Zhao, Zhengli Kang, Xiufang He, Xianfang Song.

**Methodology:** Yufan Yuan, Zhengli Kang, Xianfang Song.

**Software:** Zhengli Kang.

**Supervision:** Xiaojing Zhao, Zhengli Kang, Xianfang Song.

**Visualization:** Xiufang He.

**Writing – original draft:** Yufan Yuan.

**Writing – review & editing:** Yufan Yuan, Xiaojing Zhao, Xianfang Song.
